# The Network Pharmacology Study of Dahuang Fuzi Decoction for Treating Incomplete Intestinal Obstruction

**DOI:** 10.1155/2022/2775434

**Published:** 2022-04-28

**Authors:** Jun-feng Guo, Yu-ting Zhao, Quan-yu Du, Yuan Ren, Yan Wang, Zhen-xing Wang, Wei Jin

**Affiliations:** ^1^Chengdu University of Traditional Chinese Medicine, Chengdu, Sichuan 611130, China; ^2^Tianjin University of Traditional Chinese Medicine, Tianjin 3016172, China; ^3^Hospital of Chengdu University of Traditional Chinese Medicine, Chengdu, Sichuan 610075, China

## Abstract

**Objective:**

To explore the mechanism of Dahuang Fuzi decoction in the treatment of incomplete intestinal obstruction (IIO) based on network pharmacology and molecular docking.

**Methods:**

The chemical components of Rhubarb, Aconite, and Asarum were searched by the Traditional Chinese Medicine Systems Pharmacology database, where the possible active components were screened by oral bioavailability and drug likeness as filtering indicators. The relevant targets in the Swiss Target Prediction database were obtained according to the structure of the chemical components confirmed by the PubChem database. Disease targets of IIO were collected using GeneCards and OMIM databases. We obtained the cross-target using VENNY to capture the common targets. PPI analysis was performed on the intersection genes combined with Cytoscape 3.7.2. Gene Ontology (GO) function enrichment analysis and Kyoto Encyclopedia of Genes and Genomes (KEGG) pathway enrichment analysis were carried out by David database. The core targets and active ingredients were molecularly docked through AutoDock Vina software to predict the detailed molecular mechanism of Dahuang Fuzi decoction for treating IIO.

**Results:**

There are 45 active components in Dahuang Fuzi decoction, with 709 corresponding targets, 538 IIO targets, and 97 common targets, among which kaempferol, deltoin, and eupatin are the main active ingredients. 10 core targets were obtained by protein-protein interaction network analysis. Through GO enrichment analysis, it was found that Dahuang Fuzi decoction may be involved in biological processes such as signal transduction, anti-apoptosis, promotion of gene expression, regulation of cell proliferation, and differentiation. Besides, KEGG pathway analysis revealed that it mainly relates to PI3K-AKT signal pathway and HIF-1 signal pathway, etc. Molecular docking results showed that the active ingredients of Dahuang Fuzi decoction possess a good binding activity with the core targets.

**Conclusion:**

Dahuang Fuzi decoction may act on target genes such as TNF, IL6, AKT1, VEGFA, SRC, EGFR, and STAT3 through active ingredients such as kaempferol, deltoin, and eupatin to regulate signaling pathways such as PI3K-AKT and HIF-1 and reduce the expression of various inflammatory factors such as TNF-*α*, IL-6, iNOS, and COX-2 to play a role in the treatment of IIO.

## 1. Introduction

Intestinal obstruction (IO), one of the common surgical acute abdomen, can be divided into complete intestinal obstruction (CIO) and incomplete intestinal obstruction (IIO) according to the degree of obstruction. IIO is a condition in which the normal flow of intestinal contents is interrupted and the intestinal proximal to the obstruction becomes dilated while the distal intestinal is normal or collapsed [1], with the most common symptoms and signs being nausea, vomiting, cramping abdominal pain, obstipation, and abdominal distension [2]. IIO is a frequent cause of hospitalization, accounting for up to 20% of surgical consultations for acute abdominal pain [3], which can be attributed to a wide range of reasons from tumors and inflammatory conditions to ingested material. IIO does not reach the surgical indication, so it is generally treated conservatively by internal medicine. However, the limited therapeutic options available in Western make the IIO state unimproved for a long time, which can easily aggravate the patient's condition, and result in malnutrition, electrolyte disorder, septic shock, intestinal ischemic necrosis, and other adverse consequences [4].

Dahuang Fuzi decoction is composed of three herbs including *Radix Rhei Et Rhizome* (Rhubarb), *Aconiti Lateralis Radix Praeparata* (Aconite), and *Asari Radix Et Rhizoma* (Asarum), which was originally described in Synopsis of Golden Chamber (Jin Kui Yao Lue), a treatise on febrile and miscellaneous diseases written by physician Zhang Zhongjing in Han Dynasty, and has been widely used in China for thousands of years [5]. Nowadays, Dahuang Fuzi decoction was frequently employed to treat patients suffering from appendicitis [6], acute kidney injury [7], acute intestinal obstruction [8], and IIO [9]. At present, the clinical treatment of IIO mostly adopts a combination of Chinese and Western medicine, where Dahuang Fuzi decoction exhibited satisfying efficacy in treating patients with IIO, with less recurrence. Furthermore, clinical studies have proven that Dahuang Fuzi decoction can accelerate the recovery of patients with IIO and effectively reduce their serum TNF-*α* and IL-6 levels. Experimental studies have also demonstrated that Dahuang Fuzi decoction is capable of promoting intestinal peristalsis, reducing the damage to the epithelial cells of small intestinal mucosa, and protecting the intestinal barrier. However, the underlying mechanism of action of Dahuang Fuzi decoction for the treatment of IIO is unveiled, whose action targets, molecular mechanisms, and related signaling pathways have not been fully elaborated. Chinese herbal formulation has the characteristics of multi-component, multi-target, and multi-pathway, but the current research on action mechanisms is still limited to a single target pathway, which is not in line with the features of Chinese medicine and led to an incomplete explanation of the mechanisms.

Network pharmacology is a new discipline based on the theory of systems biology, the network analysis of biological systems, and the selection of specific signal nodes (nodes) for the design of multitarget drug molecules, which can be adopted to predict the molecular mechanism of drug action in disease and is very suitable for the multi-component, multi-target, and multi-pathway research. Molecular docking is mainly used to study intermolecular interactions and predict the binding mode and relationship. Besides, molecular docking can also be utilized for the prediction of drug and protein function. By integrating network pharmacology and molecular docking, we aimed to explore the underlying mechanism of Dahuang Fuzi decoction for the treatment of IIO in order to facilitate the condition of patients with IIO and reduce the mortality rates.

## 2. Materials and Methods

### 2.1. Collection of Active Ingredients and Target Prediction of Dahuang Fuzi Decoction

We searched Rhubarb, Aconite, and Asarum in the herb name of TCMSP (Traditional Chinese Medicine Systems Pharmacology Database and Analysis Platform) (Table 1 shows the website of the platforms used) to find multiple active compounds. Oral bioavailability (OB) represents the rate and degree of absorption of traditional Chinese medicine in the human circulatory system, while drug likeness (DL) is the similarity of compounds to known drugs. OB ≥ 30% and DL ≥ 0.18 were used to screen the active ingredients of Dahuang Fuzi decoction. The names of the compounds collected from the TCMSP database were entered into the PubChem database, and the SMILE and 3D “Standard Delay Format” (SDF) structures of the corresponding compounds were downloaded for the prediction of target genes and molecular docking. The SMILE structure of the active compound was uploaded into the Swiss Target Prediction database, and we set probability ≥ 0.1 to get the predicted target gene, which was downloaded in CSV format with all active compounds filtered and integrated using Microsoft Excel software. Predicted targets of the components were imported into UniProt for normalization and then restricted to the human species, where all retrieved target proteins were corrected to their official names.

### 2.2. Acquisition of the Common Targets of Active Ingredients and IIO

Through the GeneCards and OMIM databases, targets related to IIO were searched by entering the keyword “Incomplete Intestinal Obstruction.” VENNY was used to draw a Venn diagram of the common targets of active ingredients and IIO.

### 2.3. Construction of the “Herb-Disease-Ingredient-Target” Network

The main components of Dahuang Fuzi decoction and the common targets of active ingredients and IIO were imported into Cytoscape (3.7.2) to generate a “Herb-Disease-Ingredient-Target” network, where the nodes in the network diagram are the chemical components and targets and the correlation between components and targets is represented by edges.

### 2.4. Protein-Protein Interaction Analysis and Core Target Screening

The intersections of active ingredients and IIO targets were uploaded to the online site of STRING. The protein type was set to “Homo sapiens,” and the other parameters were set to default values. The protein interaction relationships were retrieved. The combined score from the export file was imported into Cytoscape to conduct topology analysis, build a PPI network diagram, and select the core proteins with the top 10 degree values by the cytoHubba.

### 2.5. Gene Ontology and Kyoto Encyclopedia of Genes and Genomes Pathway Enrichment Analyses

Based on the David database, GO analysis of the relevant obtained intersection target proteins was performed, involving the biological process (BP), cellular component (CC), and molecular function (MF). KEGG was selected for target pathway annotation analysis with *P* set less than 0.05, and the top 20 KEGG signal pathways were ranked according to the results in descending order of count value.

### 2.6. Molecular Docking

Molecular docking is a method for drug design by exploring the interaction and recognition between receptors and ligands, which is a theoretical simulation method that focuses on the study of intermolecular interactions and the prediction of their binding patterns and affinities. In recent years, molecular docking methods have become an important technique in the field of computer-aided drug research. AutoDock Vina is an open-source molecular docking program designed by the Scripps Research Institute for the computation of semiflexible molecular docking. AutoDock Vina utilizes a sophisticated gradient algorithm and multithreaded techniques for superior accuracy and faster prediction speed in comparison with AutoDock4. Semiflexible docking means that the conformation of the ligand molecule can be changed according to the receptor molecule and is flexible, while the receptor molecule does not change and is rigid [10]. Then, the optimization of the SDF structures of the active ingredients was accomplished by Chem3D 18.0. The corresponding number of the core target was searched in the UniProt database, and the receptor structure was downloaded in the PDB database according to the numbers. Next, the files of active compounds and ligand molecules were imported into the AutoDock tool to remove these target proteins' water molecules and add polar hydrogen to create active pockets, which were subsequently saved as PDBQT format files for later use. Molecular docking was carried out by adjusting target protein X-Y-Z coordinates and grid size and optimizing the position of protein structure-binding sites. AutoDock Vina was run to dock the treated active compound to the target protein ten times with the minimum binding energy of each docking taken as the final result. The complex was docked by PyMOL software finally.

## 3. Results

### 3.1. Collection of Active Ingredients and Target Prediction of Dahuang Fuzi Decoction

Based on the screening conditions of the compounds, 35 active ingredients were collected from the TCMSP database, among which 13 components of Rhubarb were obtained after excluding 3 components with no target, and 14 components of Aconite were acquired after excluding 7 components with no target, and 8 active components of Asarum all with corresponding targets were collected. The details are shown in Table 2. The predicted targets using Swiss Target Prediction database were compiled, resulting in 380 predicted targets of Rhubarb, 897 predicted targets of Aconite, and 693 predicted targets of Asarum. The total number of targets was 709 by combining the duplicates.

### 3.2. Acquisition of the Common Targets of Active Ingredients and IIO

**Figure 1:**
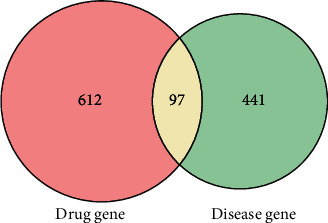
Drug-disease intersection gene Venn diagram.

The GeneCards and OMIM databases were searched with the keyword “Incomplete Intestinal Obstruction”, yielding 538 IIO targets based on the relevance score. The 97 intersecting genes of Dahuang Fuzi decoction and IIO were obtained, as shown in Figure 1.

### 3.3. Construction of the “Herb-Disease-Ingredient-Target” Network

**Figure 2:**
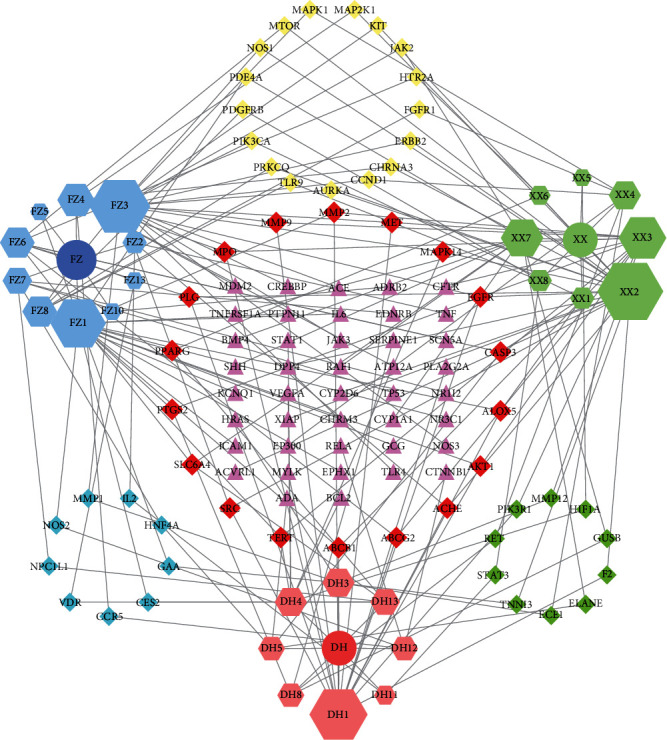
Herb-Disease-Ingredient-Target network. The size of the graph represents the degree value. The circular DH, FZ, and XX represent Rhubarb, Aconite, and Asarum, respectively. The number, chemical composition, and drug source are shown in Table 2. The red diamond represents the common target of three drugs and diseases, the sky blue diamond represents the common target of Rhubarb, Aconite, and diseases, and the green diamond represents the common target of Rhubarb, Asarum, and diseases. The yellow diamond represents the common target of Aconite, Asarum, and disease, the purple triangle represents the common target of each of the three drugs and disease, and the connecting line represents the relationship among drugs, components, action targets, and diseases.

The “Herb-Disease-Ingredient-Target” network graph created by Cytoscape 3.7.2 reflects the correspondence of the compound targets, as shown in Figure 2. The size of the nodes indicates the degree value, with higher degree values signifying a greater number of nodes connected to it and a greater regulatory role in the whole network. The active ingredients with high degree values include kaempferol, deltoin, and eupatin, which are shown in Table 3.

### 3.4. Protein-Protein Interaction Analysis

**Figure 3:**
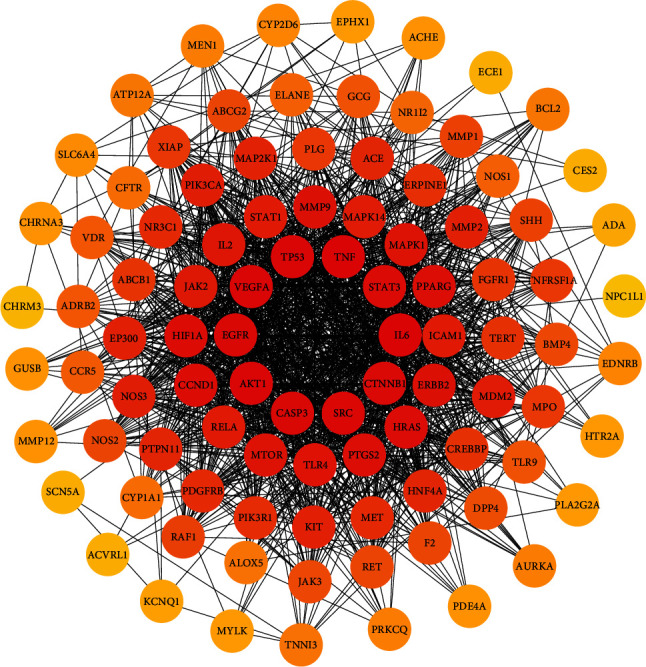
PPI network of common targets of drugs and diseases. The PPI network was constructed using the plug-in targets from the Search Tool for the Retrieval of Interacting Genes/Proteins database, which was imported into Cytoscape, and the targets were the candidates used for incomplete intentional construction treatment. Proteins are represented by nodes (colors from red to yellow illustrate the extent to which the medical targets have combined). Edges indicate protein-protein associations. The top 10 targets (hub targets) in the PPI network are ranked by degree values using the cytoHubba plug-in.

The 97 intersection targets of the predicted Dahuang Fuzi decoction and IIO were imported into the STRING database to obtain protein interaction relationships. The analysis results were topologically analyzed by Cytoscape 3.7.2, as shown in Figure 3. Nodes represent proteins, and edges represent relationships between proteins, resulting in a total of 97 nodes and 1382 edges. The colors from yellow to red represent small to large degree values, respectively. The top ten targets of degree value were filtered as core targets using cytoHubba, and according to the degree values, the key nodes, which are TNF (tumor necrosis factor), IL6 (interleukin 6), AKT1 (protein kinase), VEGFA (vascular endothelial growth factor), TP53 (tumor protein P53), SRC (nonreceptor tyrosine kinase), CASP3 (cystatin protease), EGFR (epidermal growth factor), CTNNB1 (oncogene), and STAT3 (signaling sensor and transcription activator), were selected for interactions with IIO.

### 3.5. Gene Ontology Analysis and Kyoto Encyclopedia of Genes and Genomes Pathway Enrichment Analysis

**Figure 4:**
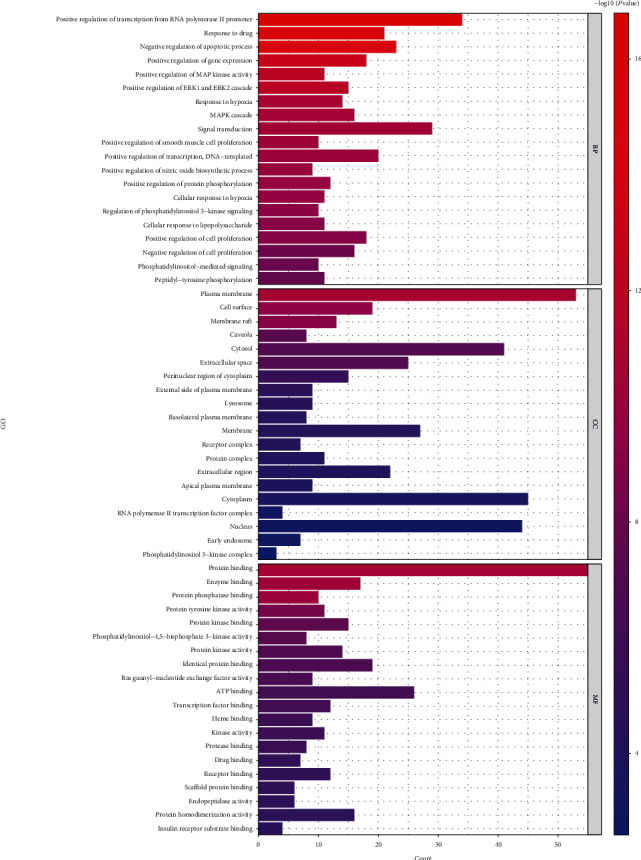
Gene Ontology functional enrichment analyses. The names of the biological processes, cellular component, and molecular function terms are distributed in the ordinate and the number of genes in the abscissa. *P* values indicate the importance of enrichment; the lower is the *P* values, the redder is the color of the graph, and the higher is the enrichment.

**Figure 5:**
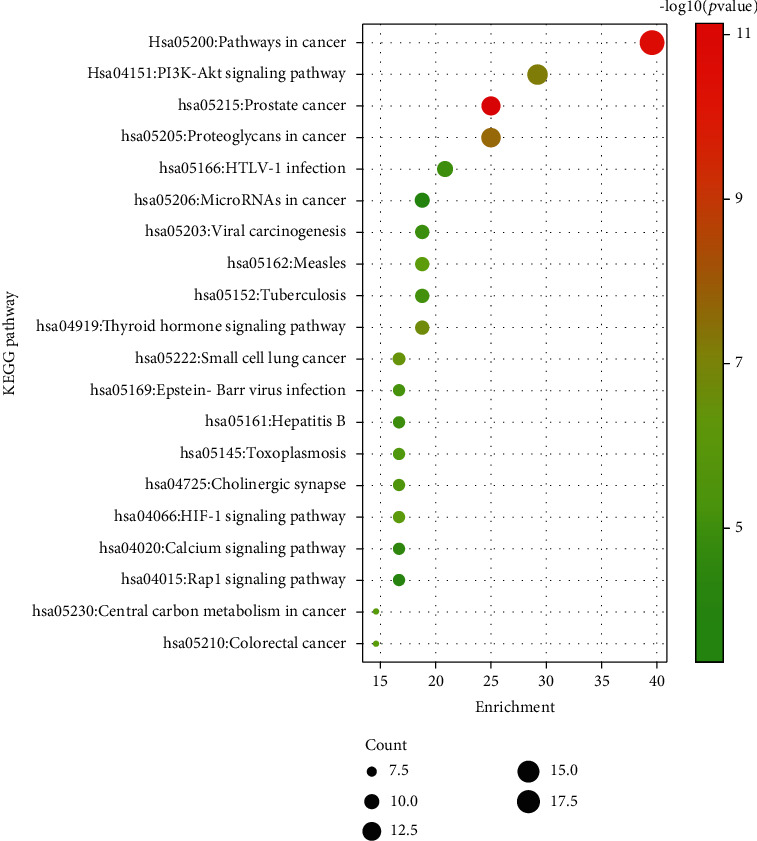
Kyoto Encyclopedia of Genes and Genomes pathway enrichment analyses. The size of the dots represents the number of genes; the larger is the dot, the higher is the number of genes in the corresponding process. *P* values indicate the importance of enrichment; the lower is the *P* values, the redder is the color of the graph, and the higher is the enrichment.

To further explore possible mechanisms of the 97 candidate targets for the treatment of IIO, the David database was used for GO enrichment analysis and KEGG pathway analysis of the candidate targets for the treatment of IIO with Dahuang Fuzi decoction. The results showed that the number of BP terms was 506, CC was 51, and MF was 92. The top 20 BPs are shown in the bar chart (Figure 4). KEGG pathway enrichment analysis was conducted by the David database and involved 111 terms (*P* < 0.05). The bubble chart (Figure 5) reflects the top 20 entries.

### 3.6. Molecular Docking

Table 4 shows the results acquired from the molecular docking software (AutoDock Vina). Processed by PyMOL software, the docked complex and “best-docked complex” show images of the best docking of the receptor and ligand. According to Table 4, we can conclude that the binding energies of the three active ingredients to the top ten ranked target gene (TNF, IL6, AKT1, VEGFA, TP53, SRC, CASP3, EGFR, CTNNB1, and STAT3) transcriptional proteins were all less than -5 kcal/mol, indicating a high affinity between the compounds and the core target genes.

## 4. Discussion

As of 2021, IIO will remain one of the most common acute abdomens, with a mortality rate of 8%~13%. According to clinical studies, the main pathogenesis of IIO is intestinal injury, inflammation, edema, nerve injury, etc. [11, 12]. The damage to the intestine can cause the release of certain cytokines and inflammatory mediators, which lead to impaired intestinal barrier function and increased intestinal mucosal permeability, thus promoting inflammatory responses [13]. Therefore, the inflammatory mediators play a pivotal role in the development of IIO, where the degree of damage and duration of IIO is closely related to the extent of inflammatory response in the intestine [14]. So, it is of great significance to provide aggressive anti-inflammatory therapy in the treatment of IIO. To further improve the condition of patients with IIO and reduce the mortality rates, this study identified the active ingredients and possible detailed molecular mechanisms of Dahuang Fuzi decoction, which can support its wider applications and further investigations in the treatment of IIO.

First of all, oral administration is the most popular mode of delivery of herbal decoction, so the oral bioavailability of the active ingredients must meet a certain standard in order to exert therapeutic effects. Therefore, the active ingredients in Dahuang Fuzi decoction were selected by TCMSP with the criteria of OB ≥ 30%, DL ≥ 0.18, and the ADME (absorption, distribution, metabolism, and excretion) parameters. Consequently, 35 active components of Dahuang Fuzi decoction were obtained, and 709 candidate targets of 35 active components were retrieved through Swiss Target Prediction. Subsequently, we constructed a Herb-Disease-Ingredient-Target network, which can reflect the relationship between numerous components and targets of the drug. As a result, three main active components of Dahuang Fuzi decoction for the treatment of IIO were identified involving kaempferol, deltoin, and eupatin. Kaempferol, as a flavonoid, can scavenge free radicals, reduce the release of inflammatory factors, and improve intestinal homeostasis. The paper published in 2020 by Yao et al. [15] revealed that kaempferol is able to suppress the levels of TNF-*α* and IL-6 as well as the activation of NF-*κ*B and has a strong antioxidant activity to remove reactive oxygen species clusters and stimulate and upregulate the expression of Nrf2, which is a key transcription factor for protecting cells from oxidative damage, and a scavenger of free radicals. Imran M et al. [16] found that kaempferol could significantly reduce the mRNA expression levels of iNOS and COX-2, thereby inhibiting the release of inflammatory factors. Kaempferol can also play a key role in antioxidation, anti-inflammation, and anti-apoptosis in a variety of tissues and organs [17]. Deltoin is a compound with significant anti-inflammatory effects in the root of Divaricate Saposhnikovia Root [18], which has not been adequately studied. Wang et al. [18] suggested that deltoin could inhibit LPS-induced macrophages expression of iNOS and COX-2, thus producing anti-inflammatory effects. Bahadır et al. [19] proposed that deltoin could exert anti-inflammatory effects by inhibiting the expression of TNF-*α*. Eupatin, a methoxyflavonoid from Artemisia, has been confirmed to have iNOS and AchE inhibitory effects. Chou et al. [20] found that eupatin significantly inhibited LPS-induced macrophage and microglia expression of iNOS and COX-2, and nitrite production, with significant anti-inflammatory effects. The level of NO is closely related to the occurrence of inflammatory diseases, whose expression is regulated by iNOS, making iNOS a key enzyme in the process of inflammation [21]. COX-2 generally has low activity in normal tissues, but it can quickly respond to some inflammatory factors such as PGE2 when exposed to external stimuli, so as to induce inflammatory cells to release chemokines, recruit inflammatory cell movement, and induce the expression of IL-6 and IL-1 in macrophages together with LSP [22]. In conclusion, all three key active ingredients, namely, kaempferol, deltoin, and eupatin, significantly inhibited the expression of iNOS and COX-2, thus exerting anti-inflammatory effects, protecting the intestine from inflammatory storm and relieving the symptoms of IIO.

Third, we retrieved 538 candidate IIO targets from the GeneCards and OMIM databases, and 709 candidate targets of Dahuang Fuzi decoction were selected through Swiss Target Prediction. We found 97 targets in common between the disease and herbs, which were considered potential targets for treating IIO. The degree of association between these genes is shown in Figure 3. The top 10 core genes include TNF, IL6, AKT1, VEGFA, TP53, SRC, CASP3, EGFR, CTNNB1, and STAT3, which play a significant role in the proliferation, migration, and apoptosis of IIO. Studies have shown that TNF-*α* and IL-6 are sensitive indicators of inflammatory response in patients with IIO, with TNF-*α* being the most critical initiator of inflammatory response in the body [23], causing a chain reaction of various damage factors, and inducing the production and release of other inflammatory factors. Various inflammatory factors interact with each other to produce an amplifying effect and chain reaction, which in turn aggravates the symptoms of intestinal obstruction and systemic pathological damage. IL-6 is a major inducer of protein synthesis in the acute phase of the human inflammatory response, which stimulates the inflammatory response by regulating the function of various inflammatory cells and intensifies the effect of inflammatory cells on inflammatory mediators, with the concentration of IL-6 in serum or plasma sensitively reflecting the degree of tissue damage [24]. Clinical studies have proven that the treatment of patients with IIO with Dahuang Fuzi decoction can accelerate patients' recovery by effectively reducing their serum TNF-*α* and IL-6 levels [25]. Intestinal compression and ischemia can stimulate the body's monocyte-macrophage system to produce excessive IL-6, TNF-*α*, and other inflammatory factors, while EGFR activation can promote the repair and regeneration of peripheral blood vessels, lymphatic vessels, and peripheral nerves and regulate various biological processes such as cell proliferation, differentiation, migration, and survival [26] to inhibit the inflammatory response, reduce pain, and relieve the symptoms of incomplete intestinal obstruction. Intestinal damage caused by IIO can pose an increase in AKT1 and VEGF gene expression levels in intestinal tissues, leading to secretion, recruitment, and infiltration of inflammatory factors. AKT is mainly involved in the PI3K-AKT signaling pathway, and its activation and expression can regulate various molecular cell physiological functions and processes, including cell metabolism, growth, proliferation, migration, apoptosis, etc. [27]. Activation of AKT upregulates the NF-*κ*B signaling pathway, and NF-*κ*B is involved in the transcriptional regulation of proinflammatory response factors [28], where VEGF upregulates multiple proinflammatory signaling pathways [29]. SRC-3 inhibits excessive activation of neutrophils in IIO, reduces neutrophil infiltration into the intestinal muscular layer, and suppresses the expression of inflammatory factors to protect the intestine from damage caused by excessive inflammatory responses and act as an immunomodulatory agent [30] in IIO. STAT3 is involved in regulating the JAK2/STAT3 inflammatory signaling pathway, which can affect the release of inflammatory cytokines to reduce intestinal inflammation in IIO [31]. JAK/STAT3 pathway is considered to be the “master switch” for the initiation of inflammatory response in the body [32]. Effective regulation of this critical signaling pathway has the potential to contain the amplifying effect of the inflammatory waterfall at an early stage of the lesion and reduce inflammatory damage in the intestine.

The target genes of Dahuang Fuzi decoction for the treatment of IIO were used to obtain an enrichment map of the GO and KEGG pathway analyses based on the David database. Figure 4 shows that BP is mainly associated with signal transduction, antiapoptosis, promotion of gene expression, regulation of cell proliferation, and differentiation. The main signaling pathways are the cancer pathway, PI3K-AKT signaling pathway, HIF-1 signaling pathway, thyroid hormone signaling pathway, calcium signaling pathway, etc. In China, the main causes of intestinal obstruction are extra-abdominal hernia, tumors, and intestinal adhesions, while the incidence of tumors is on the rise which has jumped to second place in the spectrum of causes of intestinal obstruction and is the first cause for elderly patients [33]. Dahuang Fuzi decoction alleviates the symptoms of incomplete intestinal obstruction from its etiology by inhibiting multiple cancer pathways. Activation of the PI3K/AKT signaling pathway reduces the incidence and severity of intestinal inflammation [34], whereas TNF-*α* and IL-6 induce activation of the PI3K/Akt pathway through multiple mechanisms [35]. HIF-1*α* is a subunit of HIF-1, which is upregulated in response to hypoxic stress and acts as a nuclear transcription factor, targeting and regulating the expression of downstream genes [36]. IGF-1 is a downstream target gene of HIF-1*α*, which can promote the repair of damaged epithelial cells, cellular collagen synthesis, and extracellular matrix synthesis and strengthen the intestinal mucosal barrier [37]. Therfore, Dahuang Fuzi decoction may repair the intestinal wall tissue via upregulating IGF-1 by activating HIF-1*α*. In human intestinal endothelial cells, direct interaction of the thyroid hormone nuclear receptor with the p85 subunit of PI3K leads to T3-dependent PI3K activation, resulting in phosphorylation and activation of AKT and iNOS [38], which exacerbates the intestinal inflammatory response. Dahuang Fuzi decoction attenuates the inflammatory response by regulating the thyroid hormone signaling pathway. The weakening and loss of gastrointestinal motility are the main causes of intestinal obstruction, where peristalsis of smooth muscle cells is the ultimate effector of intestinal motility, and the calcium signaling pathway is an important pathway that regulates this physiological process. When the calcium signaling pathway is blocked, Ca^2+^ does not reach the appropriate concentration, followed by the relaxation of the smooth muscle cell, thus reducing gastrointestinal motility. When the calcium signaling pathway is activated, Ca^2+^ flows into the cell through this channel in the cell membrane and promotes the contraction of smooth muscle [39]. Dahuang Fuzi decoction can promote the inward flow of calcium ions by regulating calcium ion channels, stimulating gastrointestinal muscle contraction and promoting gastrointestinal motility, improving the rhythm of intestinal contraction, and playing a regulatory role in gastrointestinal motility.

## 5. Conclusion

We hypothesize that Dahuang Fuzi decoction may act on target genes such as TNF, IL6, AKT1, VEGFA, SRC, EGFR, and STAT3 through active ingredients such as kaempferol, deltoin, and eupatin to regulate signaling pathways such as PI3K-AKT and HIF-1 and reduce the expression of various inflammatory factors such as TNF-*α*, IL-6, iNOS, and COX-2 to play a role in the treatment of IIO. In conclusion, Dahuang Fuzi decoction acts through multiple targets and pathways in the treatment of IIO, and the results of the present study provide a basis for further research on the clinical application of Dahuang Fuzi decoction in IIO and also offer guidance and reference for the next stage of study on the action mechanism of Dahuang Fuzi decoction.

## Figures and Tables

**Table 1:** Website and purpose of using the platform.

Num	Platform	Website	Purpose
1	TCMSP	https://tcmspw.com/tcmsp.php/	Search for active components of the drug
2	Swiss Target Prediction	http://www.swisstargetprediction.ch/	Search the corresponding target of active components of the drug
3	UniProt	https://www.uniprot.org/	Standardize the target names
4	PubChem	http://pubchem.ncbi.nlm.nih.gov/	Search the corresponding structure of active components of the drug
5	GeneCards	https://www.genecards.org/	Search the disease-related targets
6	OMIM	http://omim.org/	Search the disease-related targets
7	VENNY	https://bioinfogp.cnb.csic.es/tools/venny/	Screen the intersection targets of traditional Chinese medicine and diseases
8	STRING	https://cn.string-db.org/	Conduct PPI analysis
9	David	https://david.ncifcrf.gov/	Analyze the enrichment of core targets
10	PDB	https://www.rcsb.org/	Screen protein receptor structure

**Table 2:** Active components of Dahuang Fuzi decoction.

Mol ID	Molecule name	OB (%)	DL	Source	Number
MOL002235	Eupatin	50.8	0.41	Rhubarb	DH1
MOL002251	Mutatochrome	48.64	0.61	Rhubarb	DH2
MOL002259	Physciondiglucoside	41.65	0.63	Rhubarb	DH3
MOL002260	Procyanidin B-5,3′-O-gallate	31.99	0.32	Rhubarb	DH4
MOL002268	Rhein	47.07	0.28	Rhubarb	DH5
MOL002276	Sennoside E_qt	50.69	0.61	Rhubarb	DH6
MOL002280	Torachrysone-8-O-beta-D-(6′-oxayl)-glucoside	43.02	0.74	Rhubarb	DH7
MOL002281	Toralactone	46.46	0.24	Rhubarb	DH8
MOL002288	Emodin-1-O-beta-D-glucopyranoside	44.81	0.8	Rhubarb	DH9
MOL002293	Sennoside D_qt	61.06	0.61	Rhubarb	DH10
MOL002297	Daucosterol_qt	35.89	0.7	Rhubarb	DH11
MOL002303	Palmidin A	32.45	0.65	Rhubarb	DH12
MOL000358	Beta-sitosterol	36.91	0.75	Rhubarb	DH13
MOL002211	11,14-eicosadienoic acid	39.99	0.2	Aconite	FZ1
MOL002388	Delphin_qt	57.76	0.28	Aconite	FZ2
MOL002392	Deltoin	46.69	0.37	Aconite	FZ3
MOL002395	Deoxyandrographolide	56.3	0.31	Aconite	FZ4
MOL002397	Karakoline	51.73	0.73	Aconite	FZ5
MOL002398	Karanjin	69.56	0.34	Aconite	FZ6
MOL002401	Neokadsuranic acid B	43.1	0.85	Aconite	FZ7
MOL002410	Benzoylnapelline	34.06	0.53	Aconite	FZ8
MOL002416	Deoxyaconitine	30.96	0.24	Aconite	FZ9
MOL002419	(R)-norcoclaurine	82.54	0.21	Aconite	FZ10
MOL002421	Ignavine	84.08	0.25	Aconite	FZ11
MOL002423	Jesaconitine	33.41	0.19	Aconite	FZ12
MOL000359	Sitosterol	36.91	0.75	Aconite	FZ13
MOL000538	Hypaconitine	31.39	0.26	Aconite	FZ14
MOL009849	Asarinin	31.57	0.83	Asarum	XX1
MOL012141	Caribine	37.06	0.83	Asarum	XX2
MOL000422	Kaempferol	41.88	0.24	Asarum	XX3
MOL002962	(3S)-7-hydroxy-3-(2,3,4-trimethoxyphenyl)chroman-4-one	48.23	0.33	Asarum	XX4
MOL001558	Sesamin	56.55	0.83	Asarum	XX5
MOL002501	[(1S)-3-[(E)-but-2-enyl]-2-methyl-4-oxo-1-cyclopent-2-enyl] (1R,3R)-3-[(E)-3-methoxy-2-methyl-3-oxoprop-1-enyl]-2,2-dimethylcyclopropane-1-carboxylate	62.52	0.31	Asarum	XX6
MOL012140	4,9-Dimethoxy-1-vinyl-beta-carboline	65.3	0.19	Asarum	XX7
MOL001460	Cryptopin	78.74	0.72	Asarum	XX8

**Table 3:** The three active components with the highest degree value.

Molecule name	Degree	Source	Number
Kaempferol	20	Asarum	XX2
Deltoin	18	Aconite	FZ3
Eupatin	17	Rhubarb	DH1

**Table 4:** The results of molecular docking.

Target	Target structure	Compound	Compound 2D structure	Affinity (kcal/mol)	Best-docked complex
TNF	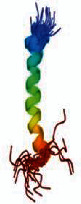	Deltoin	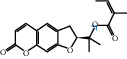	-6.0	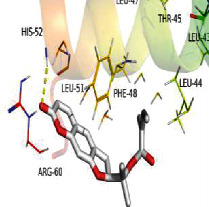
		Kaempferol	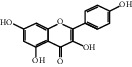	-5.1	
		Eupatin	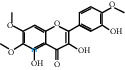	-5.3	
IL6	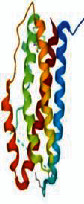	Deltoin	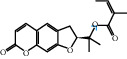	-6.7	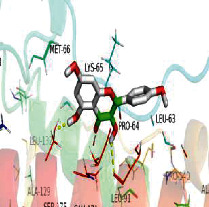
		Kaempferol	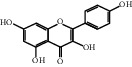	-6.8	
		Eupatin	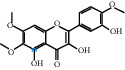	-6.1	
ATK1	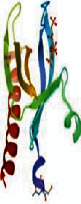	Deltoin	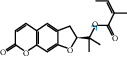	-6.1	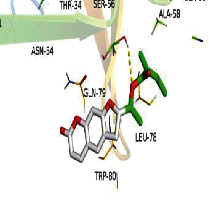
		Kaempferol	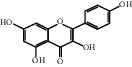	-5.9	
		Eupatin	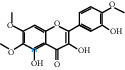	-5.3	
VEGFA	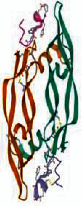	Deltoin	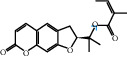	-6.3	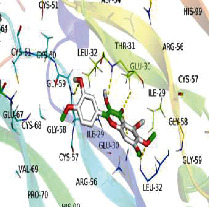
		Kaempferol	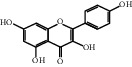	-6.2	
		Eupatin	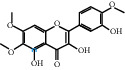	-6.4	
TP53	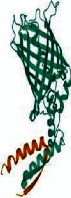	Deltoin	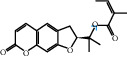	-6.1	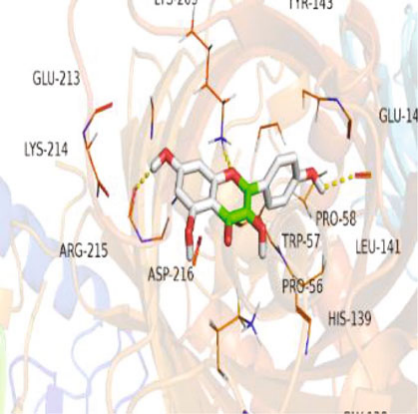
		Kaempferol	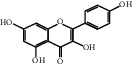	-6.3	
		Eupatin	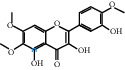	-5.3	
SRC	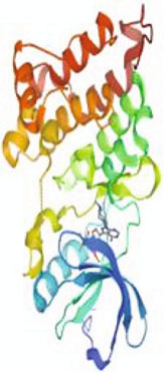	Deltoin	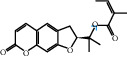	-8.6	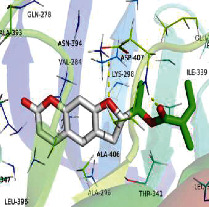
		Kaempferol	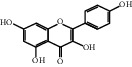	-8.1	
		Eupatin	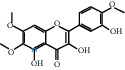	-7.2	
CASP3	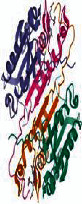	Deltoin	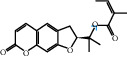	-7.4	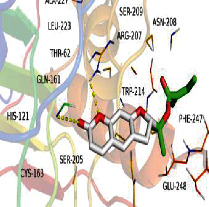
		Kaempferol	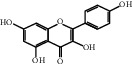	-6.0	
		Eupatin	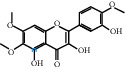	-6.1	
EGFR	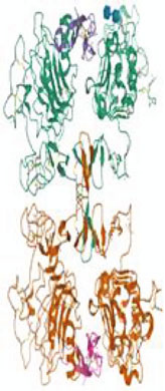	Deltoin	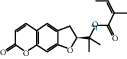	-8.0	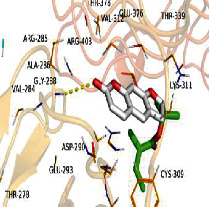
		Kaempferol	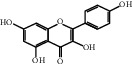	-7.7	
		Eupatin	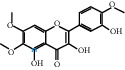	-7.1	
CTNNB1	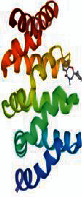	Deltoin	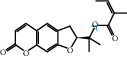	-6.2	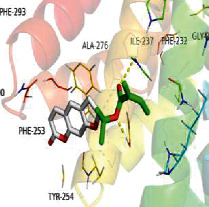
		Kaempferol	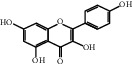	-5.4	
		Eupatin	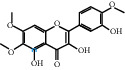	-5.8	
STAT3	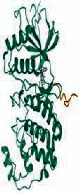	Deltoin	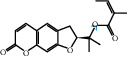	-7.2	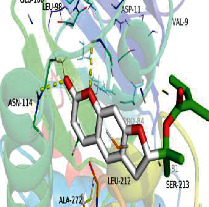
		Kaempferol	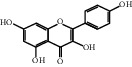	-6.7	
		Eupatin	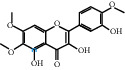	-6.4	

## Data Availability

The data used to support the findings of this study are available from the corresponding author upon reasonable request. All the data can be downloaded from the open databases mentioned in this article.
